# Psychometric Assessment of a Physician-Patient Communication Behaviors Scale: The Perspective of Adult HIV Patients in Kenya

**DOI:** 10.1155/2013/706191

**Published:** 2013-02-11

**Authors:** Juddy Wachira, Susan Middlestadt, Michael Reece, Chao-Ying Joanne Peng, Paula Braitstein

**Affiliations:** ^1^Department of Applied Health Sciences, School of Health, Physical Education, and Recreation, Indiana University, 1025 East 7th Street, Bloomington, IN 47405, USA; ^2^Department of Research, USAID-Academic Model Providing Access to Healthcare (AMPATH) Partnership, Nandi Road, P.O. Box 4606, Eldoret 30100, Kenya; ^3^Center for Sexual Health Promotion, Indiana University, 1025 East 7th Street, Bloomington, IN 47405, USA; ^4^Department of Counseling and Educational Psychology, School of Education, Indiana University, 201 North Rose Avenue, Bloomington, IN 47405-1006, USA; ^5^School of Medicine, Moi University, Nandi Road, P.O. Box 4606, Eldoret 30100, Kenya; ^6^Division of Internal Medicine and Geriatrics, School of Medicine, Indiana University, 340 West 10th Street, Indianapolis, IN 46202, USA; ^7^Regenstrief Institute, Inc., 1050 Wishard Boulevard, Indianapolis, IN 46202, USA; ^8^Division of Global Health, Dalla Lana School of Public Health, University of Toronto, 155 College Street Health Science Building, Toronto, ON, Canada M5T 3M7

## Abstract

*Introduction*. There have been no scales specifically developed to assess physician-patient communication behaviors (PPCB) in the sub-Saharan population. *Aim*. We revised an existing PPCB scale and tested its psychometric properties for HIV patients in Kenya. *Methods*. 17 items (five-point scale) measuring PPCB were initially adopted from the Matched Pair Instrument (MPI). Between July and August 2011, we surveyed a convenient sample of 400 HIV adult patients, attending three Academic Model Providing Healthcare program (AMPATH) clinics in Eldoret, Kenya. Of these 400, eight also participated in cognitive interviews, and 200 were invited to return after one week for follow-up interviews; 134 (67%) returned and were interviewed. Construct and content validity were established using an exploratory factor analysis, bivariate analyses, internal consistency, test-retest reliability and cognitive interviews. *Results*. Construct and content validity supported a one-dimensional measure of 13 PPCB items. Items assessed physicians' effort to promote a favorable atmosphere for interaction with HIV patients. Biases associated with encoding and comprehension of specific terms, such as “discussion, involvement or concerns,” were noted. Internal consistency (*Cronbach's alpha* = .81) and one-week retest reliability scores (.82) supported the reliability of the 13-item scale. *Discussion*. The revised PPCB scale showed acceptable validity and reliability in Kenya.

## 1. Introduction

Good physician-patient communication behaviors (PPCB) have been associated with patient adherence to treatment [1–6]. Patients who are more engaged with their physicians are shown to adhere to treatment and report better health outcomes [2, 5–11]. Hence, over the last decade, there has been a move to create a good interpersonal relationship between physicians and patients, enhance health information exchange, and promote congruence in treatment decisions [1–4]. Factors, such as health organization structures, health provider attitude, time spent with patient, physician-patient relationship, and provider communication skills [8–11], play an important role in defining patients' comprehension of treatment regimen. Health systems are therefore encouraged to embrace a more patient-centered approach to healthcare delivery [4, 7, 12].

Physician communication behaviors are particularly pertinent in HIV care due to the adverse health outcomes associated with inconsistent care and treatment [13, 14]. Unfortunately, there are few published empirical studies in sub-Saharan Africa that have assessed physician-patient communication dynamics within the HIV setting [15, 16]. These studies mainly explored physician-patient communication barriers and did not develop or recommend measures that could be used to assess PPCB. Therefore there is a critical need for a regional specific scale that takes into account the social and cultural dynamics, which may differ significantly from other regions. Furthermore, there is a need to explore the impact of PPCB on adherence to HIV care, considering the high rates of missed medical appointments in the region [14, 17, 18]. Our study therefore focused on Kenya, which still grapples with the challenges of HIV [19] including adherence to treatment and missed medical appointments [17, 18, 20, 21].

It is however important to note that assessing PPCB is complex because it involves a combination of behaviors and attributes that occur during a medical encounter [7, 22]. To this end, the call is for the development of appropriate instruments or further validation of existing instruments [22] in order to enhance the quality of findings. Psychometric assessments of construct validity and reliability have been widely used to determine the soundness of PPCB instruments [7, 22–25]. Unlike in developed countries where a number of PPCB scales exist [7, 22, 23, 25], there was a lack of empirically published scales for the sub-Saharan region.

We therefore used a PPCB scale from the Matched Pair Instrument (MPI) which was developed and validated in Canada [23]. This scale defined PPCB as a combination of verbal and action-related behaviors performed by physicians [23, 25]. The instrument was selected because it was drawn from several existing physician-patient measures. It comprised 19 items on a five-point Likert-type rating scale assessing two main subscales, the process and content of communication [23]. Our study assessed the psychometric properties of this scale from the patient's perspective as a primary step to developing an appropriate measure for the region. The objectives of the study were to

1. identify the factors underlying the PPCB scale;
2. determine if the PPCB scale demonstrates content validity;
3. assess the reliability of the scale, including internal consistency and test-retest reliability.

## 2. Methods

The study was conducted between July and August 2011, in three adult HIV clinics at the Moi Teaching and Referral Hospital (MTRH), located in Eldoret, Kenya. These HIV clinics were part of the USAID-Academic Model Providing Access to Healthcare (AMPATH) Partnership that provides comprehensive healthcare services to HIV patients across 65 government health facilities in western Kenya. The study protocol was approved by the Institutional Research and Ethics Committee of Moi University/Moi Teaching and Referral Hospital (Kenya) and the Committee on Protection of Human Subjects of Indiana University, Bloomington (US).

We surveyed a convenient sample of 400 HIV-positive adult (18 years and older) patients on combined antiretroviral therapy (cART) during their routine medical visits. Of these 400, eight (four men and four women) were identified to participate in cognitive interviews using the purposive sampling method. For eight days, the first man or woman enrolled in the study was invited to participate in the cognitive interviews. Furthermore, using the convenient sampling method, 200 patients out of the remaining 382 in the sample were invited to return after one week for a follow-up interview, to assess the test-retest reliability of the instrument. 

### 2.1. Patient HIV Care Process at MTRH

Typically, within the AMPATH HIV care setup, patients would be seen by a different clinician each time they visited the clinic. Trained clinicians including medical and clinical officers play a critical role in providing initial and routine HIV treatment and care. Depending on the disease progression, viral and immunity levels, and other medical considerations, HIV patients are either put on cART or closely monitored with no HIV medication. Patients receiving cART were seen by clinicians 2 weeks after initiating treatment and then monthly thereafter. Those who had not initiated cART returned every 1 to 3 months depending on their clinical status and comorbidities. During these visits patients were seen by multiple care providers, including nurses, clinicians, pharmacy technicians, nutritionists, peer outreach workers, and social workers. For new patients, clinical contact began at registration, followed by the nurse who checked vital signs. The patient also saw a peer outreach worker for documentation of locator information and then went on to see the physician/clinician. Returning patients went directly to the nurse and then followed a course similar to that established for new patients. All patients newly initiated on cART who missed a scheduled clinic visit triggered an outreach attempt within 24 hours, through either a telephone contact or a home visit conducted by trained peers. 

### 2.2. Development of Study Instrument

The study instruments included the PPCB scale from the MPI and incorporated other measures that assessed patients' socio-demographic and health characteristics. Four experts including one instrument development expert from Indiana University, two HIV patients, and two members of the AMPATH research team assessed the face validity of the instruments. This was a subjective assessment of how the instruments were presented to the experts in order to identify and address any comprehension and culturally inappropriate issues that may affect participants' responses to questions. In addition, study instruments were pretested with a convenient sample of four HIV adult patients, one week before the study commenced. Necessary revisions on the instruments were then made. The initial English versions of the instruments were translated to Swahili and back translated to ensure that both the English and Swahili versions of the study instruments were similar in content. 

### 2.3. Study Instruments

The study was conducted in four stages: (1) recruitment survey, (2) main survey, (3) cognitive interviews, and (4) follow-up survey.

*Recruitment Survey*. The instrument provided measures of specific patient characteristics including gender, whether on cART and self-reported measures of missed medical appointments and missed cART medication.

*Main Survey*. The instrument included the PPCB scale which contained 19 items on a five-point Likert-type rating scale (strongly agree, agree, neither agree nor disagree, and disagree, strongly disagree) (Table 5). Other measures included (a) socio-demographic characteristics, namely, age, marital status, tribal affiliations, education, income, mobile phone ownership, location, number of children in the household, and total number of household members; (b) patient health characteristics including self-reported measures of general health perceptions and number of white blood (CD4) cell count.

*Cognitive Interviews*. This involved an intensive one-on-one interview with respondents on the thought process of responding to PPCB items. The process aimed at eliminating measurement errors that may be associated with encoding, comprehension, judgment, recall, and reporting bias. We adapted a cognitive guide [26] that provided a framework of probes used to elicit interpretations of items on the scale. The probes included components of think aloud, comprehension retrieval, judgment, and response (Table 6). 

*Follow-Up Survey.* This included the same PPCB scale presented in the initial instrument. 

### 2.4. Data Collection

All interviews were conducted in either Swahili or English depending on participants' preferences. The majority (98%) were interviewed in Swahili, which is the Kenyan national language. Three research assistants trained by the first author were available to assist with data collection. Patients were recruited as they waited for their routine medical consultation. First, a written consent was obtained from willing participants. This was followed by the administration of the recruitment instrument (five minutes) to determine patients who were eligible for the study. Only those eligible and willing were recruited. The initial instrument (20 minutes) was then administered to all participants once they had been seen by their clinician. Separate cognitive interviews (20 minutes) were conducted 20 minutes after the identified 8 participants had completed the initial instrument. Two hundred participants were invited to return after one week for the reliability follow-up interview (10 minutes), and all returning participants were interviewed. All surveys were administered in a private room at the clinic. Patients were compensated Ksh 150 (or USD $2) for their participation. Data collected were safely stored; computer data files were secured with a well-protected password while all other data collected (paper-based and audio-recorded tapes) were stored in a secure cabinet under key and lock. 

### 2.5. Analyses

All analyses were performed using SPSS-18 statistical package. A *P* value less than .05 was considered statistically significant. Descriptive statistics, namely, mean, standard deviation, and range were performed on continuous measures of socio-demographic and health status. Percentages were provided for categorical variables. The 19 items from the PPCB scale were examined for normality and missing values. Two items, “*If a physical examination was required for your health concerns, the doctor fully explained what was done and why*” and “*Explained the lab tests needed (e.g., blood, X-rays, ultrasound, etc.)*”, were deleted because they contained missing values of greater than 47%. These items did not apply to most of the participants since HIV patients did not undergo physical examinations or tests on every medical visit. To address skewness, a logarithmic transformation was applied to the remaining 17 items. 

*Factor Analysis*. An exploratory factor analysis was performed on 17 items using the principal axis factoring (PAF) method. The number of factors extracted was based on the Kaiser rule (i.e., eigenvalues >1.0). An orthogonal varimax rotation was then performed to finalize the factor loadings. In addition, bivariate analysis such as Pearson's *r* (continuous variables), Spearman correlation (ordinal variables), and point-biserial correlation (dichotomous variables), Chi-square tests (categorical variables) and were used to test associations between the emerging subfactors and patients characteristics. These patient characteristics included socio-demographic (age, sex, marital status, education level, area of residence, and source of income) and health status (perception about health status, length in HIV care, months on cART medication, number of missed medical appointments in the past year, and missed cART medication in the past week) factors.

*Reliability*. Internal consistency of the scale was assessed using the Cronbach's alpha for all items as well as separately for items in each of the emerging factors. Item discrimination was also assessed with item-total correlation. Correlation between initial survey test scores and follow-up survey (re-test) scores was performed to determine the test-retest reliability.

*Content Validity*. Cognitive interview recordings were transcribed verbatim, translated from Swahili to English, and coded for themes. Themes identified highlighted areas of concern with the scale. 

## 3. Results

### 3.1. Patients Characteristics

A total of 400 patients enrolled and participated in the study. The mean age was 39.5 years (SD = 8.95, range 19–73) and females represented 56.5%. More than half were married (57.3%). The highest education level attained was predominately primary level (55.3%). A considerable percentage resided in an urban area (65.3%), engaged in odd jobs as a source of income (49.5%), and earned less than Ksh 1,999 (USD $25) in the past month (44.3%). Similarly, an important percentage of respondents, perceived to be in good health (45.8%), reported to have a CD4 cell count of 201 and more (61.3%). About half (49.5%) reported to have missed at least one medical appointment in the past year, and a majority (83.5%) claimed not to have missed their cART medication in the past 1 week (Table 1). The mean length of patients in HIV care was four years (SD = 2.90, range 0–13), while the mean length of those receiving cART was 42 months (SD = 31.87, range 1–156).

A greater percentage (68%) of patients were seen by male clinicians. Given that the study heavily relied on the patients' perspective, we were not able to determine which patients were seen by either a medical or, clinical officer. However based on the patient's last medical visit, the majority (89%) were seen by a different clinician. 

Of those requested to return for follow-up interviews, more than half (134 or 67%) returned after one week and participated in the interviews. There were no statistically significant differences between those who did and those who did not return for follow-up interviews in terms of age (*P* = .85), gender (*P* = .11), and previous missed appointments (*P* = .60).

### 3.2. Face Validity

Expert reviews showed that the PPCB scale was generally comprehensible, acceptable, and culturally relevant to the target population. However, experts thought that some items were a bit too general, given the cultural background of the population. Three items were highlighted: “*discussed treatment options with you*”, “*discussed next steps including any follow-up plans,*” and “*showed concerns about you as a person.*” Suggestions to simplify the items by providing more specific examples that would elicit discussion or concern were made. For example, “*Did your doctor ask how your family is fairing on?*” to capture concern. These issues were not addressed during the instrument development stage of the study because we wanted to further test the appropriateness of the items by performing a factor analysis and content validity. 

The use of a five-point response format was reported to be appropriate. However, given the relatively low education background of the population, experts suggested that detailed instructions with pictorial examples (i.e., number of oranges to indicate level of agree or disagree) be used to enhance response and eliminate any judgment or reporting biases. 

### 3.3. Content Validity

Cognitive interviews also revealed that the scale was acceptable and culturally relevant. Participants reported that they did not have any difficulty recalling what transpired in the medical observation room because the interviews were held immediately after their consultation. None of the eight patients interviewed felt that they could provide any suggestions on how to improve the items. They felt that the items were ideal based on the context of the study.

We however noted that participants interpreted the term “discussion” as the clinician asks a question and the patient responds. When a participant was asked to explain why they agreed with the statement “*discussed treatment options with you”,* they responded “*I agreed because I told him (clinician) what I was suffering from. He then told me to go for the drugs from the pharmacy.”* The same issue was also noted with the item “*involved you in decisions about your health as much as you wanted*”; a participant explained, *“He (clinician) involved me. I told him my eyes are teary in the morning. He asked if I use charcoal or firewood for cooking. I told the doctor that I use firewood and he prescribed some drugs.” *Hence, biases associated with encoding of the words “discussion” and “involvement” were evident.

### 3.4. Factor Analysis

Basing a factor analysis on the 17 PPCB items, three factors were extracted based on the Kaiser rule of eigenvalues >1. Using .45 as the cut-off point, 16 items loaded well on either one of the three factors. The item that did not load “*discussed next steps including any follow-up plans*” was deleted. Factor loadings ranged from .49 to .81. Total variance explained by the first factor (also sub-scale) was 41.0%, the second 9.6%, and the third 7.8% (Table 2). Items loading in sub-scale one mainly assessed clinicians' effort to enhance a positive atmosphere for communication. Items in subscale two focused on clinicians' effort to encourage patient participation in treatment. Finally, we could not establish what items in sub-scale three captured because they included items that would be conceptually categorized in either sub-scale one or two.

### 3.5. PPCB Subscales One and Two

Following the highlighted issues with sub-scale three and those raised in the expert reviews and cognitive interviews regarding the interpretability of items, we decided to drop the items in this category. We further performed another factor analysis on the remaining 13 items, to confirm the factor loads. Similar to the initial loadings (Table 2) the same nine items loaded on sub-scale one with a total variance explained of 46.6% and the same four items loaded on sub-scale two with a total variance explained of 9.5%. We then explored if there were any important variations in the correlations of the two sub-scales (independently) with specific patient socio-demographic and health characteristics. As indicated in Table 3, there were no significant variations in the factors associated with the two sub-scales as independent measures. This suggests that the sub-scales could be assessed as a one-dimensional measure.

### 3.6. Reliability

Internal consistency was acceptable as indicated by Cronbach's alpha of .80 for all items combined. However, this was less than commonly acceptable for items in sub-scale one (.74) (Table 4). Item-total correlations exceeded .48, which was above the .30 cutoff (Table 2). One-week re-test reliability score was high in the total scale as well as in the two sub-scales. The scores ranged from .82 to .88 (Table 4).

### 3.7. Perceptions of Clinician Communication Behaviors

Using the total scale of 13 items (sub-scales one and two) on a 5-point scale, participants gave clinicians high PPCB ratings (mean = 3.8, SD = 0.8). Higher scores meant that clinicians engaged in more communication behaviors. The mean item scores ranged from 2.88 to 4.28. The highest rated item was “*listened carefully to what you had to say*” while the lowest rated item was “*encouraged you to ask questions.*” The majority (97%) of the patients also reported that clinicians used a language that was clear and comprehendible to them. 

### 3.8. Factors Associated with Perceived PPCB

As shown in Table 3, PPCB (total scale of 13 items) was positively but weakly correlated with education level (*r* = .11) and perception about health status (*r* = .17). It was also negatively correlated with missed appointment (*r* = −.14) and missed cART medication (*r* = −.17). Participants with a higher level of education compared to those with a lower level of education gave their clinicians higher PPCB ratings. Similar findings were observed among those with a higher perception about their health status compared to those with a lower perception about their health status. On the other hand, participants who had a higher number of missed appointments and missed cART medication gave their clinicians lower PPCB ratings.

## 4. Discussion

Validating the PPCB scale in Kenya is a critical step towards developing well-tailored measures for the region. Generally, patients gave clinicians high PPCB ratings, indicating that they believed their clinicians engaged in a high number of communication behaviors. Our findings showed acceptable factors loadings that supported construct validity of 13 PPCB items. Face validity was supported by expert panel reviews and content validity by cognitive interviews. There was no evidence of recall bias following the timing of the interviews. A few modifications were recommended by experts to reduce biases associated with the encoding of specific words such as “discussion” and “involvement.” In addition, more items were needed to assess interactions between clinicians and patients. Reliability scores were found to be statistically acceptable suggesting that the scale could be consistently used to assess PPCB in Kenya.

Unlike the MPI where two sub-scales (content and process) were identified from 19 items [23], we initially found three sub-scales from 17 items. The two items that were eliminated did not apply to this population since HIV patients did not necessary undergo physical examinations or tests on every medical visit. This would be expected in the management and treatment of diseases like HIV that require long-term follow-up care. Based on a comprehensive assessment of the three sub-scales that emerged, we recommend the use of items in sub-scales one and two, as a one-dimensional measure. We noted that items in sub-scale one addressed clinicians' effort to enhance a positive atmosphere for communication. The items mainly reflected on clinicians' concern about patients' comfort with treatment. This is a fundamental component of patient-centered communication, which accounted for the highest variance, among the three sub-scales. 

Items in sub-scale two were more centered on patient involvement in treatment. Even though the variance explained was only 10%, this factor captured the core of communication, which is an interaction between the clinicians and patients. Unfortunately, these items only addressed the clinician's role in communication with no consideration of the patient's role. Hence, the scale did not present a holistic measure of communication behaviors of all parties involved. This is a weakness noted in most patient-centered communication measures [22]. Ideally measures should account for communication behaviors of each party as well as the interaction among them [22]. We therefore suggest adding a set of items that assess patients' communication behaviors during a medical encounter. 

Finally, items that loaded on sub-scale three did not make sense and accounted for the lowest variance explained (8%). Further, items in this category were general and introduced biases related with encoding of specific terms used. As previously noted for this population, there is a need to modify items that used the words “discussed,” “involved”, or “showed concern.” The interpretations of these words were generally compounded by the cultural expectation of a medical session; that is, the clinician asks the question and the patient responds. In addition, discussions about next treatment option were difficult to measure given that the majority of patients seeking medical services at the AMPATH clinics are usually there for HIV treatment, which are quite standardized and well defined. 

This points out the importance of cultural sensitivity in scale development and the value of performing cross-cultural validation of items among different populations. Furthermore, it raises the issue of social desirability which might have influenced our findings. Expectations and norms between the Kenyan and North American population may be completely different. Unlike the North American population, patients in the Kenyan population may define communication as a directive from a clinician to a patient, but not the reverse or back and forth, thus influencing their interpretation of items. It is therefore critical that we include more items that not only provide specific scenarios that exemplify these terms but are also relevant to the target population. 

Based on the concerns raised with items in sub-scale three, we dropped these items and performed a factor analysis on the remaining 13 items from sub-scales one and two. The item loadings were similar to the initial analysis confirming a two-factor loading. We further performed correlations between specific patient characteristics and the two sub-scales independently as well as collectively as a one-dimensional measure. Our findings revealed that the PPCB measure in this environment should be adopted as a one-dimensional measure. This is because there were no differences in the factors associated with the two subfactors independently. In addition, the variance explained in sub-scale two was very low highlighting the weakness of this sub-scale if adopted independently. As a next step, we propose additional regional studies that fill the gaps presented. 

Similar to findings from the adopted scale [23] internal consistency of the items was high. It was interesting to note that there was a high test-retest reliability of the PPCB. This suggests that the instrument can be consistently and reliably used to measure communication behaviors. 

It was interesting to note that patients gave their clinicians high PPCB ratings despite the fact that the majority were seen by a different clinician each time they visited the clinic. This raises the question of whether patients in this population are accustomed to a more directive style of care where the clinician makes all treatment decisions. In addition, patients of higher socioeconomic status, including education level, tend to communicate more actively and show more affective expressiveness, eliciting more information from their doctors [27]. We noted that patients with a higher education level gave better PPCB ratings than those with a lower education level. This suggests that they may have the self-efficacy to engage in communicative behaviors with their clinicians thus influencing the communication dynamics and PPCB ratings. In addition, the positive correlation of PPCB ratings with perceived health status could indicate that patients' perceptions about their health status played a significant role in influencing the PPCB ratings. 

Consistent with the reported missed appointments in this population [13, 14, 17, 21], about half had missed at least one scheduled visit in the past year. Also, patients with no missed appointments and those who had fewer missed cART medication gave their physicians higher PPCB ratings. This highlights the importance of exploring the impact of PPCB on adherence to HIV care. Previous studies in other settings have reported a strong positive association between physician communication behaviors and patient adherence to treatment [2, 5–11].

This study is not without limitations. The sample was not necessarily representative of the HIV population in Kenya. It only included those who were on cART and did not include those that were not on cART or those who had dropped out of care. Studies have shown that patients consistent in care are more accustomed to the insufficiency of their physicians and their health care in general [22]. In addition, our study only measured patients' perspective on the scale. Yet, evidence shows that physician and patients self-reported measures often do not correlate with objective ratings from the same encounters [22]. Self-reported measures were used to assess the health status of patients which may have introduced reporting biases. Furthermore, we did not explore the cognitive function of the patients that might have influenced their responses. We therefore realize that biases associated with these shortfalls may have influenced our findings. As earlier mentioned, this study is a first step to developing well-tailored instruments to assess PPCB in this population, and further studies are needed to build on our findings.

 In conclusion, our findings demonstrated that 13 items from the PPCB scale could be used in Kenya to assess clinicians' communication behaviors. We also noted that patients in this setting gave their clinicians high PPCB ratings and factors such as education level, perception about health status, missed medical appointments, and missed cART medication were statistically significantly associated with their ratings. This study is important because it documents the first validated PPCB scale in Kenya. However, we recommend further studies that build on these findings to improve on the scale and further explore the factors associated with PPCB. A combination of longitudinal studies, direct observations, and self-reports that integrate dyadic assessments could provide valuable findings. It is our conviction that an appropriate PPCB scale is a powerful tool for promoting HIV adherence in Africa.

## Figures and Tables

**Table 1:** Patient sociodemographics and health status characteristics.

	Total (*N* = 400)	
	(*n*)	(%)
Sociodemographics		

Marital status		
Yes	229	57.3
No	171	42.8
Tribal affiliations		
Lughaya	124	31.0
Kalenjin	101	25.3
Kikuyu	94	23.5
Luo	45	11.3
Turkana	14	3.5
Kisii	11	2.8
Others	11	2.8
Education Level		
None	22	5.5
Primary level	221	55.3
Secondary level	131	32.8
College/degree level	23	6.5
Area of residence		
Urban	261	65.3
Semiurban	94	23.5
Rural	45	11.3
Source of income		
Formal employment	37	9.3
Self-employed	85	21.3
Large scale farming	4	1.0
Small scale farming	33	8.3
Odd jobs	198	49.5
Family/friends	43	10.8
Income in the past 1 month		
Less than Ksh 1,999	177	44.3
Between Ksh 2,000–4,999	112	28.0
Between Ksh 5,000–10,999	70	17.5
Between Ksh 11,000–20,999	26	6.5
Between Ksh 21,000–30,999	11	2.8
Ksh 31,000 and above	4	1.0
Mobile phone		
Yes	297	74.3
No	103	25.8

Health status		

Perception of general health status		
Extremely poor	7	1.8
Poor	25	6.3
Fair	109	27.3
Good	183	45.8
Extremely good	76	19.0
CD4 cell count		
201 and more	243	61.3
200 and less	77	19.3
Do not know	78	19.5
Missed HIV clinic appointments in the past 12 months		
None	202	50.5
Once	122	30.5
Twice	55	13.8
Three times and more	21	5.3
Missed cART medication in the past 1 week		
None	334	83.5
Once	42	10.5
Twice	13	3.3
Three times and more	21	5.3

**Table 2:** Factor loading of the PPCB scale.

No.	Items	Communalities	Factor loading	Item mean	Item SD
Subscale 1 (41% variance explained)					

1	Your doctor greeted you in a way that made you feel comfortable	.43	.62	.90	.01
3	Encouraged you to express your thoughts concerning your health problems	.51	.65	.69	.12
4	Listened carefully to what you had to say	.65	.74	.23	.03
5	Understood what you had to say	.48	.63	.23	.02
17	Checked to be sure you understood everything	.50	.53	.22	.04
9	Gave you as much information as you wanted	.49	.54	.22	.04
10	Checked to see if the treatment plan(s) was acceptable to you	.37	.53	.66	.14
18	Spent the right amount of time with you	.36	.49	.23	.03
19	Overall, you were satisfied with your visit to the doctor today	.57	.65	.23	.04

Subscale 2 (10% variance explained)					

15	Involved you in decisions about your health as much as you wanted	.65	.69	.21	.04
13	Responded to your questions and concerns	.58	.68	.21	.04
12	Encouraged you to ask questions	.46	.58	.19	.05
2	Discussed your reason(s) for coming today	.34	.54	.23	.03

Subscale 3 (8% variance explained)					

11	Explained medications, if any, including possible side effects	.75	.82	.18	.05
14	Showed concern about you as a person	.65	.71	.20	.06
8	Discussed treatment options with you	.53	.66	.53	.19

Item that did not load					

16	Discussed next steps including any follow-up plans	.28	*	.59	.19

*Item did not load.

**Table 3:** Bivariate relationship between PPCB subscales and patient sociodemographic and health characteristics.

	PPCB 13 item scale					
Factors	Total scale = (subscale 1 + 2)		Subscale 1		Subscale 2	
	*r*	*P*	*r*	*P*	*r*	*P*
Sociodemographic						

Age	.00	.97	.10	.85	.01	.83
Sex	−.04	.43	−.04	.48	−.04	.46
Marital status	−.07	.19	−.09	.07	−.03	.46
Education level	.11	**.03**	.11	**.03**	.11	**.04**
Area of residence	.04	.40	−.01	.77	.08	.12
Source of income	.06	.24	.05	.29	.05	.34

Health status						

Perception of health status	.17	**.01**	.19	**.01**	.13	**.01**
Years in HIV care	−.04	.47	−.03	.54	−.03	.50
Months on cART medication	.04	.47	−.04	.48	−.04	.48
Missed medical appointments	−.11	**.01**	−.20	**.01**	−.13	**.01**
Missed cART medication	−.17	**.01**	−.15	**.01**	−.14	**.01**

**Table 4:** Internal consistency and one-week retest reliability of the PPCB scale.

Scales	No. of items	Cronbach's alpha	Item-total correlation range	1-week retest reliability
Total scale	17	.81	.65–.48	.82**
Subscale 1	9	.74	.65–.48	.80**
Subscale 2	4	.78	.65–.53	.88**

**Correlation is significant at .01.

**Table 5:** Scale from the Matched Pair Instrument assessing physician-patient communication behaviors.

No.	Items	SA	A	NAD	D	SD
		5	4	3	2	1
1	Your doctor greeted you in a way that made you feel comfortable					
2	Discussed your reason(s) for coming today					
3	Encouraged you to express your thoughts concerning your health problems					
4	Listened carefully to what you had to say					
5	Understood what you had to say					
6	If a physical examination was required for your health concerns, the doctor fully explained what was done and why					
7	Explained the lab tests needed (e.g., blood, X-rays, ultrasound, etc.)					
8	Discussed treatment options with you					
9	Gave you as much information as you wanted					
10	Checked to see if the treatment plan(s) was acceptable to you					
11	Explained medications, if any, including possible side effects					
12	Encouraged you to ask questions					
13	Responded to your questions and concerns					
14	Showed concern about you as a person					
15	Involved you in decisions about your health as much as you wanted					
16	Discussed next steps including any follow-up plans					
17	Checked to be sure you understood everything					
18	Spent the right amount of time with you					
19	Overall, you were satisfied with your visit to the doctor today					

NB: SA: strongly agree, A: agree, NAD: neither agree nor disagree, D: disagree, and SD: strongly disagree.

**Table 6:** Examples of cognitive probes.

Cognitive process	Cognitive probes
Think aloud	How did you go about answering that question? Tell me what you are thinking? I noticed you hesitated before you answered—what were you thinking about? How easy or difficult did you find this question to answer? Why do you say that?

Comprehension	What does the term X mean to you? What did you understand by X?

Retrieval	How did you remember that? Did you have a particular time period in mind? How did you calculate your answer?

Confidence judgment	How well do you remember this? How sure of your answer are you?

Response	How did you feel about answering this question? Were you able to find your first answer to the question from the response options shown?

Source: adapted from [26].

## References

[1] Ong LML, de Haes JCJM, Hoos AM, Lammes FB (1995). Doctor-patient communication: a review of the literature. *Social Science & Medicine*.

[2] Zolnierek KB, Dimatteo MR (2009). Physician communication and patient adherence to treatment: a meta-analysis. *Medical Care*.

[3] Makoul G, Krupat E, Chang C-H (2007). Measuring patient views of physician communication skills: development and testing of the Communication Assessment Tool. *Patient Education and Counseling*.

[4] Frist WH (2005). Health care in the 21st century. *The New England Journal of Medicine*.

[5] Bakken S, Holzemer WL, Brown MA (2000). Relationships between perception of engagement with health care provider and demographic characteristics, health status, and adherence to therapeutic regimen in persons with HIV/AIDS. *AIDS Patient Care & STDs*.

[6] Wilson IB, Kaplan S (2000). Physician-patient communication in HIV disease: the importance of patient, physician, and visit characteristics. *Journal of Acquired Immune Deficiency Syndromes*.

[7] Levinson W, Lesser CS, Epstein RM (2010). Developing physician communication skills for patient-centered care. *Health Affairs*.

[8] Beer L, Fagan JL, Valverde E, Bertolli J (2009). Health-related beliefs and decisions about accessing HIV medical care among HIV-infected persons who are not receiving care. *AIDS Patient Care & STDs*.

[9] Johnson MO, Chesney MA, Goldstein RB (2006). Positive provider interactions, adherence self-efficacy, and adherence to antiretroviral medications among HIV-infected adults: a mediation model. *AIDS Patient Care & STDs*.

[10] Tobias C, Cunningham WE, Cunningham CO, Pounds MB (2007). Making the connection: the importance of engagement and retention in HIV medical care. *AIDS Patient Care & STDs*.

[11] Haskard Zolnierek KB, Dimatteo MR (2009). Physician communication and patient adherence to treatment: a meta-analysis. *Medical Care*.

[12] El-Sadr WM, Abrams EJ (2007). Scale-up of HIV care and treatment: can it transform healthcare services in resource-limited settings?. *AIDS*.

[13] Fox MP, Rosen S (2010). Patient retention in antiretroviral therapy programs up to three years on treatment in sub-Saharan Africa, 2007–2009: systematic review. *Tropical Medicine and International Health*.

[14] Brinkhof MWG, Dabis F, Myer L (2008). Early loss of HIV-infected patients on potent antiretroviral therapy programmes in lower-income countries. *Bulletin of the World Health Organization*.

[15] Langlois-Klassen D, Kipp W, Rubaale T (2008). Who’s talking? Communication between health providers and HIV-infected adults related to herbal medicine for AIDS treatment in western Uganda. *Social Science & Medicine*.

[16] Watermeyera J, Penna C (2008). ‘They take positive people’ : an investigation of communication in the informed consent process of an HIV/AIDS vaccine trial in South Africa. *Critical Inquiry in Language Studies*.

[17] Braitstein P, Katshcke A, Shen C (2010). Retention of HIV-infected and HIV-exposed children in a comprehensive HIV clinical care programme in Western Kenya. *Tropical Medicine & International Health*.

[18] Ochieng-Ooko V, Ochieng D, Sidle JE (2010). Influence of gender on loss to follow-up in a large HIV treatment programme in western kenya. *Bulletin of the World Health Organization*.

[19] KDHS KDHS Kenya Demographic and Health Survey 2008-2009.

[20] Merten S, Kenter E, McKenzie O, Musheke M, Ntalasha H, Martin-Hilber A (2010). Patient-reported barriers and drivers of adherence to antiretrovirals in sub-Saharan Africa: a meta-ethnography. *Tropical Medicine & International Health*.

[21] Geng EH, Nash D, Kambugu A (2010). Retention in care among HIV-infected patients in resource-limited settings: emerging insights and new directions. *Current HIV/AIDS Reports*.

[22] Epstein RM, Franks P, Fiscella K (2005). Measuring patient-centered communication in Patient-Physician consultations: theoretical and practical issues. *Social Science & Medicine*.

[23] Campbell C, Lockyer J, Laidlaw T, MacLeod H (2007). Assessment of a matched-pair instrument to examine doctor-patient communication skills in practising doctors. *Medical Education*.

[24] Bieber C, Müller KG, Nicolai J, Hartmann M, Eich W (2010). How does your doctor talk with you? Preliminary validation of a brief patient self-report questionnaire on the quality of physician-patient interaction. *Journal of Clinical Psychology in Medical Settings*.

[25] Kenny DA, Veldhuijzen W, Weijden TVD (2010). Interpersonal perception in the context of doctor-patient relationships: a dyadic analysis of doctor-patient communication. *Social Science & Medicine*.

[26] Collins D (2003). Pretesting survey instruments: an overview of cognitive methods. *Quality of Life Research*.

[27] Suarez-Almazor ME, Conner-Spady B, Kendall CJ, Russell AS, Skeith K (2001). Lack of congruence in the ratings of patients’ health status by patients and their physicians. *Medical Decision Making*.

